# The Crosstalk Between Tumor-Associated Macrophages (TAMs) and Tumor Cells and the Corresponding Targeted Therapy

**DOI:** 10.3389/fonc.2020.590941

**Published:** 2020-11-03

**Authors:** Zhe Ge, Shuzhe Ding

**Affiliations:** ^1^ School of Physical Education & Health Care, East China Normal University, Shanghai, China; ^2^ Key Laboratory of Adolescent Health Assessment and Exercise Intervention of Ministry of Education, East China Normal University, Shanghai, China

**Keywords:** tumor, tumor-associated macrophages (TAMs), tumor microenvironment, immune suppression, therapeutic target

## Abstract

Tumor microenvironment (TME) is composed of tumor cells and surrounding non-tumor stromal cells, mainly including tumor associated macrophages (TAMs), endothelial cells, and carcinoma-associated fibroblasts (CAFs). The TAMs are the major components of non-tumor stromal cells, and play an important role in promoting the occurrence and development of tumors. Macrophages originate from bone marrow hematopoietic stem cells and embryonic yolk sacs. There is close crosstalk between TAMs and tumor cells. With the occurrence of tumors, tumor cells secrete various chemokines to recruit monocytes to infiltrate tumor tissues and further promote their M2-type polarization. Importantly, M2-like TAMs can in turn accelerate tumor growth, promote tumor cell invasion and metastasis, and inhibit immune killing to promote tumor progression. Therefore, targeting TAMs in tumor tissues has become one of the principal strategies in current tumor immunotherapy. Current treatment strategies focus on reducing macrophage infiltration in tumor tissues and reprogramming TAMs to M1-like to kill tumors. Although these treatments have had some success, their effects are still limited. This paper mainly summarized the recruitment and polarization of macrophages by tumors, the support of TAMs for the growth of tumors, and the research progress of TAMs targeting tumors, to provide new treatment strategies for tumor immunotherapy.

## Introduction

Macrophages, the dominant cell type in the tumor milieu, account for ~30–50% of the tumor tissue mass, been referred to as TAMs. Degree of TAMs tumor infiltration is intimately related to the poor prognosis of a series of tumors (1). Macrophages are classified into nonpolarized M0 macrophage, classically activated M1 macrophages, and alternatively activated M2 macrophages, which are further sub-categorized into M2a, M2b, M2c, and M2d based on the stimulation of different cytokines (2, 3). There is close crosstalk between TAMs and tumor cells. For example, TAMs are abundant in many cancers. Due to the influence of various signal factors secreted by tumor cells, they are characterized by an immunosuppressive M2-like phenotype that affects the tumor microenvironment and promotes tumor growth and metastasis by secreting a variety of substances (4).

## Origin and Recruitment of TAMs

### Origin of TAMs

It is traditionally believed that macrophages are released from the bone marrow as immature monocytes and migrate to the tissues after circulation, eventually differentiating into resident macrophages, including Kupffer cells in the liver, alveolar macrophages in the lung, and osteoclasts in the bone (5). However, the main tissue-resident macrophage population, including liver Kupffer cells, alveolar, spleen, and peritoneal macrophages, is derived from the yolk sac precursors and is not dependent on monocytes in the blood (6). TAMs are derived from Ly6C+CCR2+ monocytes in the circulation, which are derived from bone marrow hematopoietic stem cells. Then Ly6C+CCR2+ monocytes are engaged into tumor tissue and differentiated into TAMs (7). In addition, tissue-resident macrophages may also promote tumor growth by switching to TAMs (8). These results suggest that TAMs can be temporarily recruited from the blood circulation or directly transformed from the resident macrophages in tissue where the tumor is located.

### Recruitment of TAMs

#### Tumors Recruit Macrophages by Secreting Cytokines

**Figure 1:**
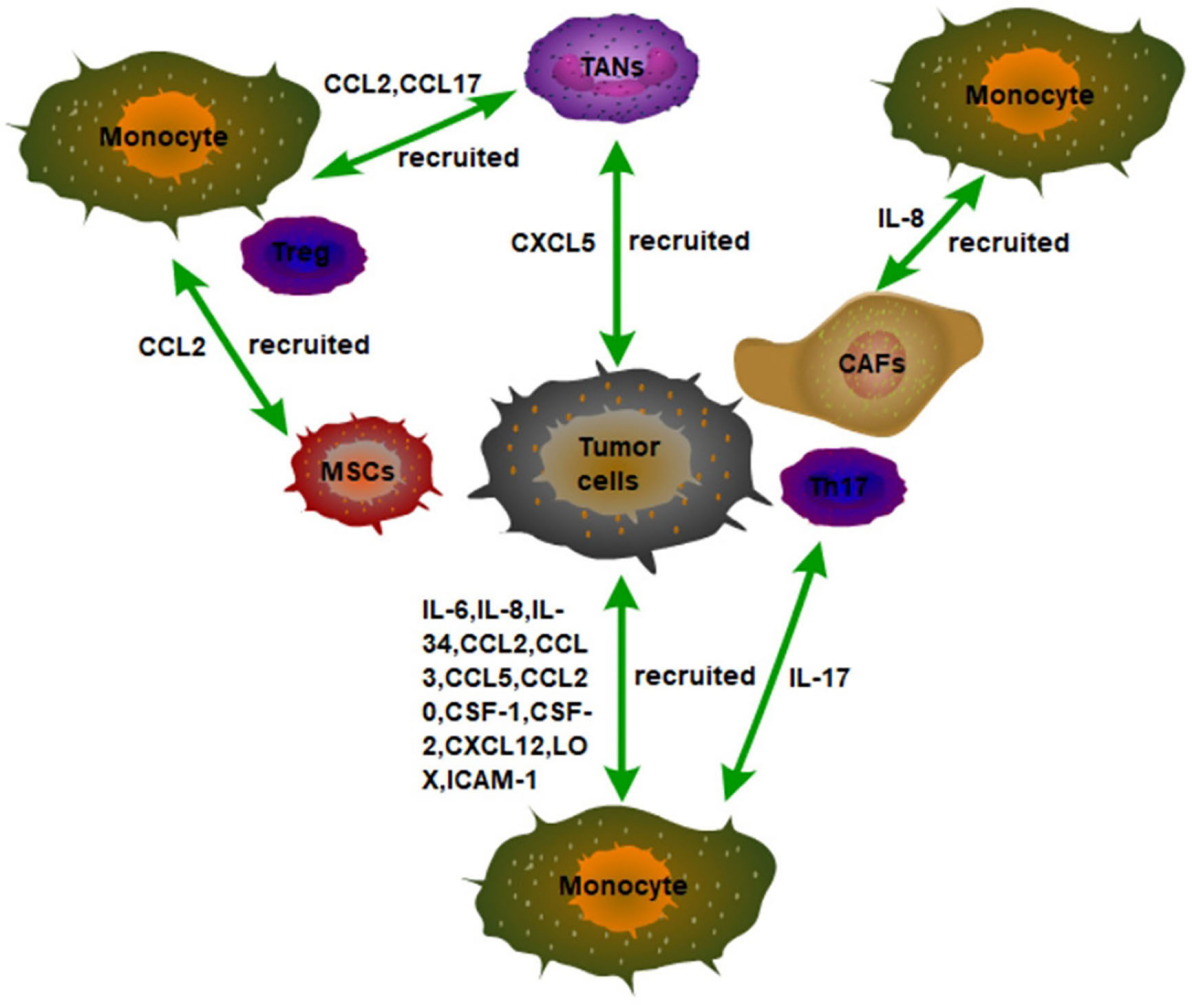
The recruitment of monocytes in the TME in tumor tissues. Tumor cells secrete a variety of inflammatory factors and chemokines to recruit monocytes to infiltrate tumor tissues, such as IL-6, IL-8, and IL34. In addition, non-tumor stromal cells in tumor tissues can also recruit monocytes to infiltrate tumors by secreting inflammatory factors and chemokines. For example, IL-8 secreted by CAFs, IL-17 secreted by Th17, and CCL2 secreted by MSCs recruit monocytes to infiltrate tumor tissues.

Tumor cells can secrete some signal molecules and interleukins to recruit macrophages (**Figure 1**). First, it has been found that the expression of colony stimulus factor 1 (CSF-1) and interleukin-6 (IL-6) in non-small cell lung cancer (NSCLC) is closely associated with the infiltration of TAMs in tumor stroma and the progression of lung cancer (9). Indeed, tumor cells can recruit macrophages into tumor tissues through CSF1, the main chemokine, and function regulator of macrophages. It is found that CSF1 overexpression in colon cancer cells is associated with macrophage infiltration, and colon cell-derived CSF1 promotes macrophage recruitment and increases IL-8 production bynbsp;macrophages. Furthermore, IL-8 secreted by macrophages, in turn, activates the protein kinase C (PKC) signaling pathway of colon cancer cells, which enhances the production of CSF1 in colon cancer cells (10), indicating that the interaction between tumor cells and TAMs forms a vicious cycle, thus promoting the recruitment of macrophages in tumor tissues. It is worth noting that hypoxia inducible factor-1 (HIF-1)-dependent production of CSF-1 in tumor cells recruits TAMs with hypoxia (11), which also means that improving the TME with hypoxia is beneficial to prevent the recruitment of TAMs. Secondly, the expression of hippo pathway effector protein (YAP) in hepatocellular carcinoma cells results in the recruitment of TAMs by secreting IL-6 (12). Adipose tissue can also recruit macrophage by secreting IL-6 (13). The expression of IL-6 in THP-1 cells enhances its own migration and infiltration into tissues (14). It is worth noting that the recruited TAMs can also secrete high levels of IL-6 (15), further recruiting macrophages to infiltrate the tumor tissue and form a vicious circle. Therefore, it is speculated that increased IL-6 levels in tumor tissues may lead to the infiltration of macrophages in tumor tissues. Also, lung tumor tissue recruits RAW264.7 macrophages and primary peritoneal macrophages to migrate through the high expression of IL-17, the process mediated by IL-17RA and IL-17RC expressed on TAMs (16). IL-34, the newly discovered cytokine in recent years, shares common features with CSF-1. It can be considered with CSF-1 as twin cytokines that are functionally related. In fact, IL-34 expressed by osteosarcoma cells, is regulated by IL-1β and TNF-α, can also recruit macrophages into tumor tissues (17). Finally, the high expression of CSF2 in breast cancer is associated with more CC chemokine ligand 18 (CCL18)+ macrophage infiltration, epithelial-mesenchymal transition (EMT), enhanced metastasis, and reduced patient survival (18). These results indicate inflammatory TME may be the key for the recruitment of macrophages.

Tumor cells can also secrete chemokines to recruit macrophages to infiltrate the tumors. Tumor cells can also recruit chemokine (C-C motif) receptor 2 (CCR2)-expressing monocytes into tumor tissues by secreting CCL2 (19). This indicates that the CCL2/CCR2 axis participates in the recruitment of TAMs into tumor tissues. Besides, it has been observed that PBMCs in NSCLC patients released higher levels of CCL2 after LPS stimulation compared with non-smokers, supporting the involvement of CCL2 in NSCLC biological processes (20). As we all know, CCL20 is deemed to be produced by intestinal epithelial cells, especially in response to stress. It has been found that colon cancer cells recruit monocytes to infiltrate tumor tissues by secreting CCL20 to bind with the monocyte receptor CCR6 in mouse model of colon cancer (15). Phyllodes tumors of the breast promote malignant progression by secreting CCL5 to recruit macrophages associated with repolarized tumors (21). However, the transfer inhibitor raf kinase inhibitor (RKIP) can regulate TAM recruitment by blocking high mobility group AT-hook 2 (HMGA2), leading to decreased expression of various macrophages chemokines, including CCL5. It is worth noting that the expression of RKIP in tumor cells is decreased in TNBC, which leads to the increase of CCL5 secretion to promote the recruitment of macrophages, thus enhancing the invasion of tumors (22). This also shows that targeted recovery of RKIP expression in TNBC to reduce the secretion of CCL5 in tumor cells is an important strategy to reduce macrophage infiltration. Indeed, in the mouse PDX (patient-derived xenografts) model of human malignant phyllodes tumor, intraperitoneal injection of maraviroc, CCR5 inhibitor, or CCL5 neutralizing antibody prevents the recruitment of monocytes to tumors and dramatically suppress tumor growth (21).

Activation of chemokine receptor CXCR4/CXCL12 signaling is also involved in TAMs recruitment of melanoma (23). Moreover, the invasion of TAMs in tumor tissues and the high expression of CXCL4 in NSCLC cells is intimately related to the development of NSCLC and the higher rate of lymph node metastasis (24). It has been found that glioma cells with PTEN gene deletion can activate transcription factor YAP1, which can lead to a large amount of LOX excretion. Then, LOX recruits macrophages to infiltrate into glioma cells by binding to β1 integrin on macrophages, which secretes SPP1 to support the growth of gliomas (25).

High expression of transcriptional factor Sox2 in breast cancer cells activates nuclear factor of activated T cells (NFAT), signal transducer and activator of transcription 3, (STAT3), and NF-κB signalings to secrete chemokines CCL3 and intercellular adhesion molecule-1 (ICAM-1), which recruit TAMs into the tumor microenvironment and promote tumor metastasis (26). Also, it has been found that the high expression of galectin-3 in tumor cells recruits M2 macrophages to infiltrate tumors and generate blood vessels in mouse melanoma and Lewis lung cancer models (27), but the specific mechanism remains unclear.

#### Recruitment of Macrophages by Stromal Cellular Compartment in TME

Other cells in the tumor microenvironment can also recruit macrophages into the tumor (**Figure 1**). For example, tumor cells can also recruit macrophages through tumor-associated neutrophils (TANs). Mechanically, CXCL5 derived from hepatocellular carcinoma (HCC) cells is the strongest effector of neutrophil migration under hypoxic conditions. Then, the TANs that are distributed in the HCC stroma, but not in tumor cells or adjacent non-tumor hepatocytes recruit macrophages and Treg cells into HCC by secreting CCL2 and CCL17 to promote the HCC progression (28). Furthermore, the interaction of macrophages, fat cells, and tumor cells in the microenvironment of breast cancer promotes the recruitment of macrophages in tumors and further promotes the development of breast cancer. Specifically, leptin and lauric acid secreted by adipocytes and CCL2 secreted by breast cancer cells together enhance the chemotaxis of macrophages. In addition, leptin also decreases the proportion of M1 TAMs (29). These results also mean that targeting adipocytes in TME may become a new idea for tumor immunotherapy.

Mesenchymal stromal cells (MSCs) are a non-hematopoietic pluripotent progenitor cell derived from bone marrow, capable of differentiating into a variety of cell types, including chondrocytes, adipocytes, and osteocytes. MSCs are an important component of the tumor microenvironment. MSCs can also be recruited into tumors in a CCR2-dependent manner. Moreover, TNFα-pretreated BM-MSCs mimic MSCs from tumors in their chemokine production profile and ability to promote tumorigenesis of lymphoma, melanoma, and breast carcinoma (30). In addition, CAFs recruit macrophages into tumors *via* IL-8/CXCR2 pathway (31). These results indicate that the inflammatory tumor environment is conducive to macrophage recruitment and tumor growth promotion. In addition, liver tumor-initiating cells (TICs) can recruit macrophages to sustain their growth. It has been found that TICs can recruit M2-type macrophages at the early stage of tumorigenesis. Mechanistically, TICs recruit M2-type macrophages for infiltration through activation of YAP, which induces the expression of CCL2 and CSF-1 (32). Therefore, targeting YAP in tumors may be able to slow tumor growth by reducing the recruitment of TAMs.

## Polarization of TAMs in Tumor Microenvironment

Macrophages can be divided into M1-type macrophages and M2-type macrophages according to their functions. M1 macrophages produce inflammatory cytokines such as IL-1, 6, 12, and 23, TNF-α, ROS, and NO. However, M2 macrophages produce IL-10, TGF-β, VEGF, and matrix metalloproteinase 9 (MMP9), and express argininase-1 (ARG-1), scavenger receptors (CD163 and CD204), and C-type lectin (CD301) (33). In fact, TAMs are characterized by an immunosuppressive M2-like phenotype (4). In the presence of interferon-γ (IFN-γ) and lipopolysaccharide (LPS), monocytes differentiate into M1 macrophages. However, monocytes differentiate into M2 macrophages in the presence of CSF-1, interleukin-4, IL-13, glucocorticoid, IL-10, and in the presence of immune complexes induced jointly with IL-1R or TLR ligands (34). After tumor cells recruit macrophages into tumor tissues, in order to avoid being swallowed by macrophages, they can induce M2-type polarization of macrophages in the following ways.

### Interleukins and Chemokines

It has been observed that monocytes are recruited into tumors and then differentiated into TAMs through IL-4 and IL-13 induction (35). IL-4 and IL-13, mainly derived from Th2 cells, promote M2-type polarization of macrophages through activation of STAT6 signaling (36). Importantly, tumor cells also secrete IL-4, IL-10 (37, 38), and IL-10 can also induce M2-type polarization of macrophages (39). Thus, tumor cells can induce M2-type differentiation of macrophages by secreting IL-4 and IL-10. In addition, IFN-γ knockout mice show accelerated tumor growth and M2-type TAMs during urethane-induced lung cancer. However, lung tumor growth is inhibited in IL-4R knockout mice and TAMs phenotype presents M1-type (40). These results also indicate that IFN-γ and IL-4 play an antagonistic role in the differentiation of TAMs and that targeting IL-4 in the TME may contribute to lung cancer treatment. Indeed, it has been found that targeting the elevated IL-4 in the TME also alters inflammation in the tumor microenvironment, reducing the generation of immunosuppressive M2 macrophages and myeloid-derived suppressor cells (MDSCs), which enhances anti-tumor immunity and delays tumor progression (41). Moreover, tumor-derived CSF-1 and IL-4 synergistically induce M2-type polarization of macrophages (42). Except for of the above, myeloma cells can also stimulate macrophage proliferation and TAMs polarization by overexpressing chemokines CCL2, CCL3, and CCL14 (43). Snail expressed tumor cells not only recruited macrophages by secreting cytokines such as CCL2, CCL5, and IL-6, but also secretes tumor-derived exosomes (TEXs) which contains miR-21 to induce M2-type polarization of macrophages (44).

### TGF-β

Transforming growth factor β (TGF-β) secreted by tumor cells can also induce M2-type polarization of macrophages. Mechanistically, interleukin-1 receptor associated kinase-M (IRAK-M), an inactive serine/threonine kinase, is mainly expressed in macrophages and a robust negative regulator of TLR signaling. TGF-β secreted by tumor cells induces the expression of IRAK-M in macrophages and promotes the polarization of macrophages toward M2-type, thereby promoting the tumor (45). TGF-β can also induce M2-macrophage polarization by up-regulating Snail expression through smad2/3 and PI3K/AKT signaling pathways (46).

### Other Signal Molecules

**Figure 2:**
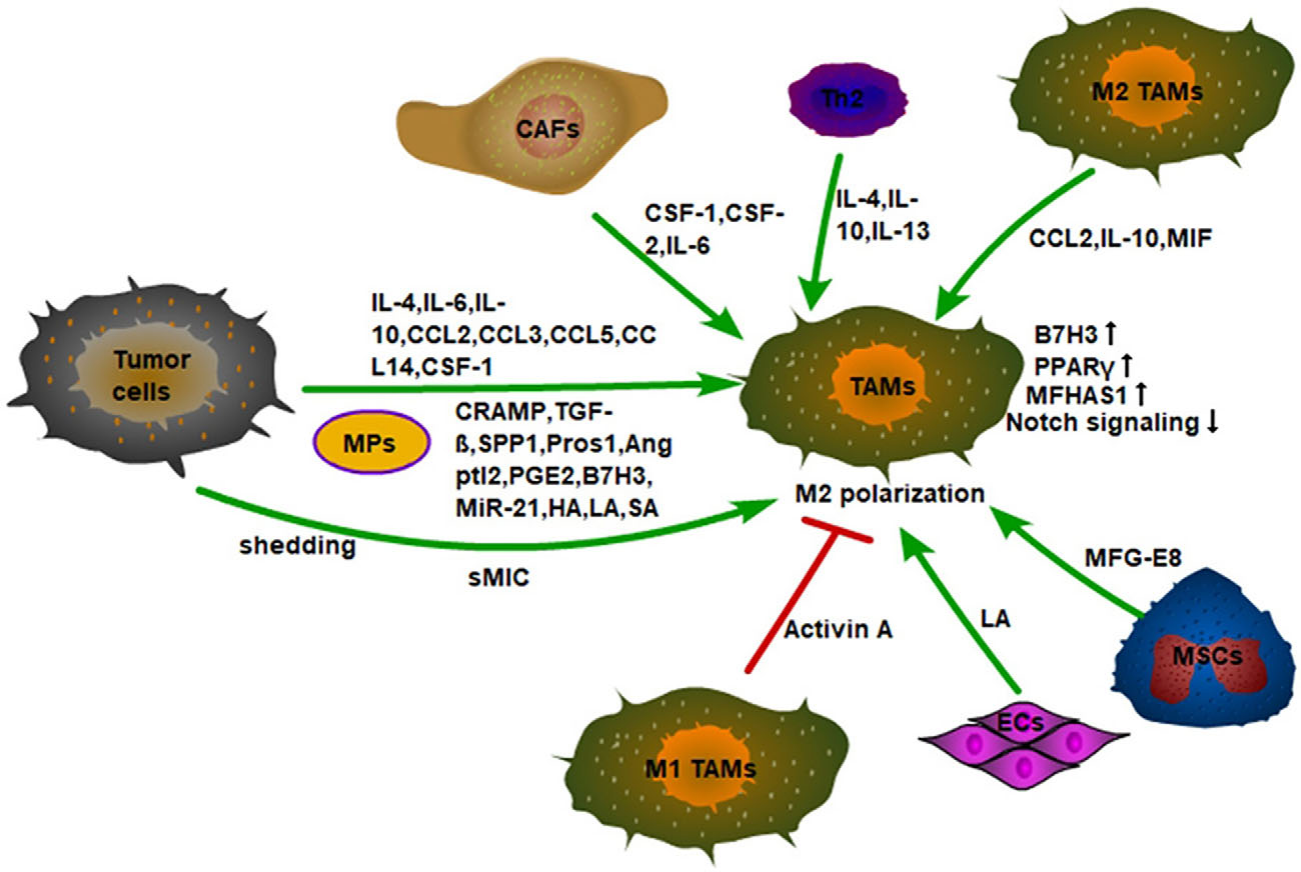
Monocytes recruited into tumor tissue are polarized into M2 macrophages by a variety of cytokines in the TME. First, tumor cells secrete inflammatory factors, chemokines, and signal molecules to promote the M2 polarization of TAMs, such as IL-4, CCL2, TGF-β, etc. Secondly, tumor cells also promote the M2 polarization of TAMs by shedding sMIC. Finally, the non-tumor stromal cells in the TME also induce M2-type polarization of macrophages. For example, CSF-1, CSF-2, and IL-6 secreted by CAFs; IL-4, IL-10, IL-13 secreted by Th2 cells, and MFG-E8 secreted by MSCs. In addition, Activin A secreted by M1 TAMs can also inhibit the M2 polarization of macrophages. ECs also promote the M2-type polarization of macrophages by secreting LA.

Tumor cells can also induce M2-type polarization of TAMs through a variety of other signal molecules (**Figure 2**). For example, tumor cells can release vesicles into the extracellular space to be absorbed by macrophages. These special subcellular vesicles are called MPs, which are between 100 and 1,000 nanometers in diameter and contain nuclear histones, caspases, microRNAs, and DNA. With the absorption of MPs, TAMs polarize into M2-type to accelerate tumor progression (47). In addition, the expression of human leucine leucine 37 (LL-37) and its mouse homologous antimicrobial peptide-related AMP (CRAMP) in both human and mouse prostate cancer is positively correlated with tumor progression. Mechanistically, the CRAMP derived from prostate cancer also mediates M2-type polarization of macrophages (48). Furthermore, lung adenocarcinoma cells secrete phosphoprotein 1 (SPP1) to induce the expression of PD-L1 in M2 macrophages and enhance the M2-polarized effect of macrophages, thus promoting tumor progression (49).

It has been found that the expression of angiogenin-like protein 2 (Angptl2) is significantly elevated in NSCLC cells, which is positively correlated with TAM infiltration, tumor size, and poor patient survival. NSCLC cells promote the polarization of M2 in TAMs by secreting Angptl2 and promote the progression of non-small cell lung cancer (50). Tumor cells also utilize Tyro3, Axl, and Mer receptor tyrosine kinases to reduce inflammation and innate immune responses. It has been found that tumor cells can secrete protein S (Pros1), a Mer/Tyro3 ligand, to suppress M1 polarization and lower anti-tumor immune response (51). PGE2, highly expressed in lung cancer tissues, induces M2-type polarization of macrophages by secretion (16). Furthermore, B7-H3, a member of the B7 family, is expressed in some types of human cancer and plays a significant role in the development of tumors. Elevated expression of B7-H3 in lung cancer tissues of NSCLC patients is found to be significantly associated with shorter overall survival (52). In addition, the elevated expression of B7-H3 in hepatocellular carcinoma is also positively correlated with the number of TAMs. Mechanically, HCC cells can induce M2-type differentiation of monocytes by secreting B7-H3 and inducing the expression of B7-H3 in monocytes, thereby stimulating the STAT3 signaling pathway (53). Importantly, cancer cells can utilize protease-mediated shedding strategies to produce soluble MIC (sMIC) to escape host immune recognition. Furthermore, sMIC also promotes MDSCs amplification by activating STAT3 and causes macrophages to be more inclined to a more immunosuppressive phenotype (54), suggesting that sMIC targeting is a target for cancer immunotherapy. It has also found that tumor tissue hypoxia leads to a large upregulation of sialic acid (SA), which is transported to bind with CD45 of MDSCs to upregulate the activity of CD45 protein tyrosine phosphatase (CD45PTP) and suppress the phosphorylation of STAT3, thus promoting the differentiation of MDSCs to TAMs (55). Thus, it is worth noting that whether the activity of STAT3 is beneficial to the differentiation of M2 macrophages still needs to be elucidated. Furthermore, the extracellular matrix (ECM) component hyaluronic acid (HA) can be produced by tumor cells, which is associated with increased tumor progression. HA released by ovarian cancer cells induces the M2 phenotype of TAMs by promoting macrophage cell-cholesterol effervescent (56).

Intermittent hypoxia significantly promotes the metastasis of Lewis lung cancer (LLC) and increased CD209+ macrophage infiltration in the primary tumor tissues. Mechanistically, hypoxia and IL-6 can promote the m2-like phenotype deflection of the tumor from M1-to enhance the metastasis of LLC. Further studies have shown that the M2 polarization of the macrophage caused by hypoxia depends heavily on the activation of its ERK signaling (57). Malignant fibrous histiocytoma amplified sequence 1 (MFHAS1) is a predictive oncoprotein that shows tumorigenic activity *in vivo*. It has been found that with the progress of CRC, the expression of MFHAS1 in TAMs increases gradually. Further studies show that CRC cells could induce MFHAS1 expression in macrophages, and subsequently promote TAMs M2 polarization by activating STAT6 and KLF4 to accelerate CRC progression. In addition, they found that the activation drive of PPAR polarizes macrophage M2 through MFHAS1-mediated activation of STAT6 and KLF4 signaling (58). Indeed, ovarian tumor stem cells (OCSCs) promote M2 polarization of macrophages by increasing PPARγ expression and inhibiting NF-κB expression (59). This also suggests that inhibit PPARγ signaling in macrophages may reverse M2-type polarization.

### The Polarization of Macrophages by Stromal Cellular Compartment in TME

M2-type macrophages can convert M1-type macrophages to M2-type macrophages by secreting CCL2 in TME. It is found that after TLR stimulation, CSF-2 polarized macrophages (M1-type macrophages) release large amounts of TNF-α and IL-6, and CSF-1 polarized macrophages (M2-type macrophages) produce low levels of pro-inflammatory cytokines and high levels of IL-10. CCL2 is highly expressed by macrophages under the action of CSF-1, while CCR2 is only expressed by CSF-2 polarized macrophages. CCL2 enhances LPS-induced IL-10 production, while CCL2 blockade leads to increased expression of M1-polarization-related genes, and decreased expression of M2-related gene in macrophages. In fact, CCR2-deficient macrophages show an M1-skewed polarization profile at the transcriptomic level and exhibit a high expression of pro-inflammatory cytokines (TNF-α, IL-6) under the stimulation of LPS (60). Furthermore, a large amount of IL-10 produced by TAMs can inhibit the production of self-IL-12, which facilitates the maintenance of TME (61). Macrophage migration inhibitor (MIF) released by macrophages also promotes M2-type polarization in melanoma (62). Moreover, it has been found that malignant tumor cells, such as glioblastomas and melanomas, can recruit mesenchymal stem cells from surrounding tissue or from the circulation into tumor tissue to sustain their growth. Then, the MFG-E8, a powerful angiogenic factor secreted by mesenchymal stromal cells can promote M2-type polarization of macrophages and promote tumor angiogenesis (63). In addition, circulating tumor cell CTC can regulate M2-type polarization of TAMs to increase tumor invasiveness, angiogenesis, and immunosuppression (64). It is to be noted that M1-type macrophages can in turn affect the polarization of M2-type macrophages. It has been observed that activin-A secreted by M1-type macrophages can promote the pro-inflammatory phenotype of macrophages and inhibit the acquisition of the M2-type through smad2/3 phosphorylation (65).

CSF-1 and CSF-2 have different polarization effects on macrophage polarization. CSF-1 co-acts with NF-κB and upregulates c-Jun expression to induce M2-type polarization in macrophages (66). Interestingly, CSF-2 can induce M1-type polarization in macrophages, while CSF-2 and IL-6 derived from tumor-associated fibroblasts (CAFs) can synergistically induce the M2-type phenotype of TAMs (67). The specific mechanism remains unclear. Furthermore, the concentration of lactic acid in the TME can also regulate the polarization of macrophages. In tumor cells, glycolysis is strongly enhanced to meet the high ATP demand of these tumor cells (68). It has been found that the energy metabolism of endothelial cells ECs is mainly dependent on glycolysis, and ECs-derived lactic acid can cause macrophages to be polarized into M2-type. However, the loss of phosphofructokinase-2/fructose-2,6-bisphosphatase isoform 3 (PFKFB3), the glycolytic regulator, reduces lactate secretion by ECs and inhibits M2-type macrophage. In addition, MCT1 knockout inhibits M2-type macrophage differentiation (69). This suggests that the production of lactic acid in the tumor microenvironment can promote M2-type differentiation of macrophages. In addition, the presence of a large number of tumor blood vessels in tumor tissues can provide nutrients for tumor cells. Not only can tumor cells produce lactic acid through glycolysis, but also their vascular endothelial cells can produce lactic acid to polarize TAMs into M2-type. The M2-type macrophages can further promote the formation of blood vessels in tumor tissues and these form a vicious circle. In addition, CAFs can increase ROS content in monocytes by secreting CSF-1, to induce M2-type differentiation of macrophages (70).

### Notch Signaling

TAMs terminal differentiation is dependent on RBP-J, a DNA-binding protein, which serves as the central transcriptional regulator of the Notch signaling pathway (71). In addition to the regulation of macrophage polarization by the above methods, the tumor also promotes M2-type polarization by inhibiting the Notch signaling of macrophages. For example, M2-like TAMs have a low level of activation of the Notch pathway in mouse tumor models, while activation of the Notch signaling in macrophages promotes M1-type polarization and enhances the anti-tumor ability of macrophages. Further studies found that RBP-J-deficient macrophages show TAM phenotype, and RBP-J-mediated Notch signaling promotes M1 polarization of macrophages through SOCS3 (72). It is worth noting that although the Notch signaling promotes the differentiation of monocyte into TAMs, the Notch signaling can promote the pro-inflammatory polarization of differentiated TAMs toward M1, which suggests that targeting to enhance the expression of RBP-J of TAMs and enhancing Notch signaling to promote the M1 polarization of TAMs may have potential for tumor treatment.

However, other studies have shown that RBP-J is the key to the M2 polarization of macrophages (73). The specific mechanism of these paradoxes is still unclear. In addition, IL-37 can also inhibit the Notch signaling pathway. In fact, IL-37 inhibits M1 macrophage polarization by suppressing the Notch1 and NK-κB pathways (74). However, it has been found that IL-37 is almost not expressed in non-small cell lung cancer tissues, but is highly expressed in adjacent normal tissues (75). In addition, the expression of IL-37 in liver cancer tissues is also decreased, and its expression level is negatively correlated with tumor size (76). These results suggest that tumor cells may not rely on IL-37 to induce M2-type polarization of macrophages.

### The Tumor Inhibits Its Phagocytosis by Binding to the TAMs Receptor

Macrophages can engulf and destroy tumor cells to prevent tumor growth. With the exception for inducing the M2 polarization of macrophages, subtle tumor cells can also inhibit their phagocytic activity by binding directly to the macrophages’ receptors. CD47 is recognized as a key protein expressed on the surface of many cancer cells, allowing them to evade innate immune surveillance and its overexpression is a common feature of leukemia cells and solid tumors. CD47 on tumor cells binds to and activates the signal regulatory protein-α (SIRPα), an inhibitory protein expressed on the surface of macrophages, to initiate a signal cascade and inhibit the phagocytic activity of macrophages (77). Additionally, recent studies have also discovered new targets for tumor cells to express “don’t eat me” signals. In addition to CD47, tumor cells also highly express CD24 molecules to achieve immune escape. CD24 can bind to the inhibitory receptor sialic-acid-binding Ig-like lectin 10 (Siglec-10) highly expressed on TAMs, thereby inhibiting the phagocytic activity of TAM (78). Besides, MHC class I on tumor cells directly protects them from macrophage attack through binding to the inhibitory receptor LILRB1 on the surface macrophages, which is up-regulated by tumor cells (79). Also, tumors also utilize the PD-L1/PD-1 axis to suppress the phagocytosis of TAMs. It has been found that PD-1 is expressed in TAMs in both mice and humans. PD-1 expression in TAM increases with tumor progression in mouse cancer models and primary human cancers. Importantly, this correlates negatively with phagocytic potency against tumor cells (80).

## Mechanism of TAMs Promoting Tumor Progression

TAMs can promote tumor progression through a variety of pathways. For example, TAMs secrete TGF-β, vascular endothelial growth factor (VEGF), fibroblast growth factor 2 (FGF2), FGF10, fibroblast growth factor receptor 2 (FGFR2), and several MMPs to support tumor growth and immune protection (81). Furthermore, it is found that patients with cancers which include pancreas, lung, undifferentiated thyroid, and gallbladder with high TAM density have a lower survival rate (82). In fact, depletion of TAMs slows the growth of urethane-induced lung cancer in mice (83). In addition, the maintenance of TAMs polarization is dependent on the presence of tumor (84), which suggests that tumor cells can maintain the M2-type polarization of TAMs. Tumor tissues are composed of varying numbers of cancer cells and stromal cells, in which the number of macrophages expressing CD204 is associated with lung adenocarcinoma invasion (85). Indeed, the specific location of TAMs in tumor tissues is closely related to tumor progression. An increase in CD204 positive TAMs in the tumor stroma is found to be associated with a poor prognosis of lung adenocarcinoma, rather than the number of TAMs in the tumor islets or alveolar cavity (86). In addition, increased expression of CD163+ TAMs and CXCL12 in tumor stroma (TS) and invasive tumor margin (TM) is intimately associated with tumor progression in GC (87). These studies indicate that the number of TAMs in tumor stroma plays a vital role in promoting tumor progression.

### TAMs Promote the Development of Tumors

TAMs in lung tumor tissue also promote lung tumor formation by the activation of NLRP3 inflammasomes that produce IL-1α and IL-1β (88). TAMs also stimulate the expression of IL-1, IL-6, and IL-8 in lung cancer cells through TLR signaling, serving to maintain the inflammatory microenvironment of the tumor and promote the development and progression of lung cancer (89). Indeed, it has been found that infiltrating macrophages secrete inflammatory mediators CCL2, IL-1α, IL-6, and tumor necrosis factor-α (TNF-α) to promote the proliferation of colon cancer cells (15). Furthermore, tumor-derived HSPs activate TLR4 signaling in TAMs to produce cytokines that are beneficial to tumor growth, such as TNF-α and VEGF. Furthermore, TNF-α secreted by TAM leads to the activation of NF-kB in tumor cells. Activation of NF-kB pathway in tumor cells prevents tumor cell death and enhances tumor cell invasion (90).

In fact, there is a large number of inflammatory cell infiltrations in tumors, among which IL-17 produced by Th17 cells and IL-23 produced by TAMs can promote tumor progression. This process may be activated by microbial products in tumors (91). Indeed, recent studies have found a large number of bacteria in tumor tissues (92), which means that inflammation caused by microorganisms in tumors is intimately related to the progression of cancer. How to target and regulate these tumor microorganisms may be the key to the future treatment of tumors.

IL-6 secreted by TAMs encourages the development of HCC through phosphorylation of STAT3 in HCC cells (93). Moreover, with the progression of lung cancer, the high expression of IL-10 in TAMs in NSCLC is associated with poor overall survival, and the expression of IL-10 in lung cancer cells is not changed (94), indicating that IL-10 secreted by TAMs during cancer progression is the key to promote cancer progression. Importantly, IL-10 elevation in TME is intimately associated with TAMs ERK1/2 signaling activation. It is found that the MyD88-dependent pathway in TAMs is selectively blocked. However, LPS and Poly (I:C) can activate downstream ERK-1/2 MAPK activation by TLRs receptors on TAMs independent of MyD88 signal, resulting in phosphorylation of the IL-10 histone promoter region, thereby increasing the expression of IL-10 of TAMs, a process independent of p38MAPK activation. Considering that LPS can induce the activation of p38MAPK and ERK-1/2 activation in normal macrophages, this phenomenon may be specific to TAMs. Additionally, abrogation of ERK1/2 activation significantly reduces IL-10 production in TAM. Moreover, activation of ERK-1/2MAPK leads to enhanced induction of IRAK M in TAM, which negatively regulates the MyD88 signaling pathway, further promoting the immunosuppression function of TAMs. Furthermore, there are also endogenous TLR ligands in tumor sites (95). This also means that these endogenous tumor TLR ligands may maintain the pro-tumor function of TAMs by activating ERK1/2 signaling downstream of the TLR signaling.

IL-6 has long been considered a mediator of tumor cell proliferation by binding to IL-6 receptors (IL-6R) (96). Indeed, IL-6 secreted by TAM acts on glioma cells and promotes 3-phosphoinositide dependent protein kinase 1 (PDPK1) mediated phosphoglycerate kinase 1 (PGK1) phosphorylation (T243), which changed the affinity between PGK1 and the substrate, promoting the activity of the glycolysis direction of PGK1, and the aerobic glycolysis, cell proliferation and tumor growth of glioma cells (97). In mouse and human neuroblastoma cells, TAMs are found to increase STAT3 activation in neuroblastoma cells, and up-regulates MYC oncogenes, a process associated with IL-6 independent expression. However, inhibition of STAT3 activation significantly retards TAMs mediated tumor growth in subcutaneous neuroblastoma in mice (96), which suggests that IL-6 is not the key to the growth of neuroblastoma and may be related to the type of tumor.

Tumor-derived extracellular vesicles (TEVs) include RNA and a variety of cytokines such as TGF-β, activated Src, Wnt3, HIF1α, in which the process also associates with tumor progression. Indeed, studies have shown that TAM can also transfer tumor-derived extracellular vesicles (TEVs) to surrounding stromal cells to make stromal cells become CAF-like cells and forming a pro-tumor microenvironment to promote tumor progression (98).

### Tumors Utilize TAMs to Promote Metastasis

TLR4, the receptor mainly expressed on macrophages, its signaling plays an active role in the process of chronic inflammation. Activation of TLR4 on M2-polarized TAMs stimulates an increase in the IL-10, which promotes the EMT of pancreatic cancer cells, specifically in increased the morphology of fibroblasts, up-regulated the expression of mesenchymal markers Vimentin and Snail, and increased the proliferation, migration, and proteolytic activities of MMP 2 and MMP9 in pancreatic cancer cells. M2-polarized TAMs co-culture with pancreatic cancer cells reduce the expression of E-cadherin, an epithelial marker. In turn, pancreatic cancer cells increased TLR4 expression in M2-polarized TAMs (99). Furthermore, M2-polarized macrophages promote HCC migration and EMT through the TLR4/STAT3 signaling pathway (100). These results suggest that TLR4 is an important target for tumor immunotherapy. Also, IL-4 produced by tumor cells not only promotes M2-type polarization of macrophage, but also promotes tumor growth and invasion by enhancing the cathepsin protease activity in TAMs (38).

COX-2 is overexpressed in all metastatic cancers. It has been found that the high expression of COX-2 in breast cancer TAMs enhances the migration and invasion of breast cancer cells. Mechanistically, the overexpression of COX-2 in TAMs activates the AKT pathway in breast cancer cells by releasing IL-6 and PGE2, thereby inducing the expression of MMP-9 and promoting EMT in breast cancer cells (101). In addition, IL-6 secreted by macrophages can up-regulate COX-2 expression and PGE2 secretion in lung cancer cells, and then COX-2 and PGE2 further promote lung cancer cell metastasis by inducing EMT through β-catenin transposition from the cytoplasm to nucleus (102), which indicates that the high expression of COX-2 in TAMs can also up-regulate the COX-2 in tumor cells through the secretion of IL-6 to promote the process of tumor metastasis. Indeed, TAMs have been proven to promote tumor cell migration and invasion by up-regulating COX-2 and MMP9 expression in osteosarcoma (OS) cells, promoting phosphorylation of STAT3 and inducing epithelial-mesenchymal transformation (EMT) (103). Trigger receptor (TREM)-1, a member of the hyperimmunoglobulin family, is primarily expressed in monocytes/macrophages and is highly expressed in colon, liver, and lung cancer tissues. Moreover, it is found that the expression of TREM-1 in tumors is closely related to tumor invasiveness of liver cancer and lung cancer. Mechanistically, lung cancer cells could induce increased expression of TREM-1 in macrophages through COX-2 signaling (104). These findings also mean that targeted inhibition of COX-2 signaling in tumor tissues may be the key to inhibit tumor metastasis.

TGF-β also causes EMT in tumor cells (105). The mechanism is that TGF-β1/Smad signaling directly activates the expression of EMT transcription factors, such as ZEB1, ZEB2, Snail, Slug, and Twist (106). TGF-β secreted by TAMs can stimulate the expression of SOX9 in lung cancer cells by stimulating the c-Jun/smad3 signaling, promote its EMT, and thus promote the proliferation, migration, and invasion of tumors. Therefore, TGF-β/SOX9 axis may represent an effective therapeutic target for lung cancer (107). Besides, fucosyltransferase IV (FUT4) and its synthetic tumor sugar antigen Lewis Y (LeY) are severely elevated in various solid tumors and play an important role in tumor invasion and metastasis. Importantly, FUT4/LeY is essential in cytoskeletal remodeling and EMT and the density of TAMs is intimately correlated with E-cadherin and LeY levels in lung adenocarcinoma. Moreover, M2 macrophages can promote the expression of FUT4/LeY through TGF-β1/Smad2/3 signaling, which mediates the development of EMT in lung adenocarcinoma by Ezrin phosphorylation (108). Tumor cells also promote TAMs secretion of TGF-β, for example, colon cancer cells with high expression of RBP-J secrete CXCL11 to promote TAMs secretion of TGF-β. Further studies show a negative correlation between RBP-J expression and E-cadherin expression, which are beneficial to the metastasis of colon cancer cells (109). ZEB1 in TAMs, a TGF-β downstream transcription factor, induces the expression of CCR2, MMP9, and CCL2 in cancer cells to accelerate tumor growth (110). Moreover, E2F3, a downstream signaling molecule of CSF-1, has been found to increase expression of E2F3 in prostate cancer, ovarian cancer, and lung cancer, and the expression of E2F3 in TAMs can promote lung metastasis of tumors (111). Overexpression of FOXQ1 has been reported in a variety of cancers, including lung cancer cells, pancreatic ductal adenocarcinoma, and the transition of intestinal epithelial cells from normal to adenoma and adenocarcinoma in APC mice. TGF-β has been proven to promote FOXQ1 expression to induce EMT. In addition, FOXQ1 directly binds to the promoter region of E-cadherin to inhibit E-cadherin expression (112). Indeed, studies have found that TAMs can induce the expression of FOXO1 protein in gastric cancer cells, thus promoting EMT, invasion, and metastasis of tumor cells (113), which indicates that FOXQ1 repression in tumor cells may be the key to inhibiting tumor metastasis.

CCL18 is mainly secreted by M2-TAMs and promotes cancer progression in a variety of human cancers. High expression of CCL18 in TAMs has been found to be associated with lymph node metastasis, distant metastasis, and poor prognosis in NSCLC patients. Mechanistically, CCL18 increases the invasion of NSCLC by binding to the Nir1 in NSCLC, which induces ELMO1-dependent cytoskeletal recombination through RAC1 activation (114). Moreover, mesenchymal breast cancer cells induce macrophages to present the TAM phenotype by GM-CSF. Similarly, CCL18 derived from TAMs induces cancer cell EMT to form a positive feedback loop (18). Furthermore, the TAMs in breast cancer produce large amounts of CCL18, which promotes breast cancer metastasis through the PITPNM3 receptor (115). In addition, the expression of CCL18 in tumor cells is also associated with enhanced invasion and migration. For example, it has been found that the high expression of CCL18 in tumor cells promotes invasion and migration of ovarian cancer cells by activating their own mTOR signaling (116). Notably, lung cancer cells can induce vimentin expression and EMT in non-cancerous receptor cells by secreting exosomes, leading to their migration, invasion, and proliferation (117), which suggests that tumor cells can also induce peripheral cell EMT to promote tumor metastasis and invasion.

### TAMs Promote the Cancer Stemness

M2-type macrophages promote cancer stem cell (CSC)-like properties by stimulating the JAK1/STAT1/NF-κB/Notch1 pathway in NSCLCs by secreting IL-10 (118). Furthermore, MUC1 in various types of cancer is a poor prognostic indicator. However, TAMs also secrete MUC1 to enhance the expression of CSC-related and inflammatory genes in lung cancer cells, such as CD133, SOX2, and NF-κB, thus promoting the generation of lung cancer stem cells (LCSCs) (119). Indeed, studies have found that the number of TAMs is positively correlated with the density of CSCs in the peripheral areas of human HCC. Moreover, TGF-β secreted by M2-type TAMs can promote the cancer stem cell-like properties via inducing EMT (120). Also, IL6 produced by TAMs promotes CSCs expansion in hepatocellular carcinoma (121). In addition, PGE2 levels are associated with colon CSC markers (CD133, CD44, LRG5, and SOX2) in human colorectal cancer samples. Further studies show that PGE2 activates NF-κB by signaling EP4-PI3K and EP4-mitogen-activated protein kinase, which induces proliferation and metastasis of colorectal cancer stem cells (122). In addition, the stemness of glioma cells can in turn recruit microglia cells. Glioma stem cells (GSCs) are the key to tumor drug resistance and heterogeneity. In the GBM study of glioblastoma, it is found that the high expression of CLOCK in GSCs could maintain the stemness of GSCs, which promotes the expression of chemokine OLFML3 as a transcription factor, thus accelerating the infiltration of microglia into GBM, but it did not affect the recruitment of macrophages, and the mechanism remains unclear (123).

### TAMs Promote Tumors EscapeImmune Surveillance

TAMs can also promote tumor cells to escape immune surveillance through the following multiple ways. First, TAMs induce the expression of B7-H4 on the surface of lung cancer cells, so that lung cancer cells can escape T cell immune recognition and destruction (124). Secondly, IFN-γ is not always effective as an anti-tumor agent. Studies have concluded that it can also promote tumor cells to evade immune surveillance. IFN-γ secreted by TAMs can induce the expression of PD-L1 in lung cancer cells through JAK/STAT3 and PI3K/AKT signaling pathways, thereby promoting tumor progression (125). Furthermore, TGF-β secreted by TAMs in Malignant pleural effusion (MPE) can increase the expression of Tim-3, PD-1, and CTLA-4 on the surface of T cells and reduce the production of IFN-γ and granzyme B to inhibit T cell cytotoxic activity in MPE (126). Moreover, TAM-derived TGF-β in MPE can induce CCL22 expression through c-Fos, which promotes the recruitment of regulatory T cells (Treg). Then, the Treg secretes high levels of IL-8 to further induce TGF-β production from TAMs, thus which forms a vicious circle, and leads to the formation of an immunosuppressive microenvironment in MPE (127). Finally, in addition to the IFN-γ mentioned above, TAMs promote the expression of PD-L1 in tumor cells by secreting IL-10 (128). Intriguingly, tumor cells can induce the expression of PD-L1 on the surface of TAMs. Indeed, tumor cells can secrete phosphoprotein 1 (SPP1) to induce the expression of PD-L1 in TAMs (49). Additionally, peripheral blood monocytes with low initial levels of PD-L1 up-regulates the expression of PD-L1, after incubation with primary tumor cells from OSCC patients, which correlates with the increased IL-10 levels in the TME (129). The increase of IL-6 in the TME also enhances the expression of TAMs PD-L1. These studies indicate that improving the inflammatory TME may be the key to reducing PD-L1 expression and reversing the immunosuppressive TME.

### TAMs Promote the Formation of Blood and Lymphatic Vessels in Tumor Tissues

TAMs also promote the production of blood and lymphatic vessels in tumor tissue. It has been found that matrix metalloproteinase 9 (MMP9) secreted by TAMs promotes the migration of tumor cells by promoting the migration of endothelial cells, which promotes angiogenesis (130). In addition, there is a cross-talk between TAM and lung cancer cells, in which the CCR2-CCL2 and CX3CR1-CXCL1 signals are the basis for lung cancer growth and metastasis. Specifically, knockout of CCR2 and CX3CR1 in mice can shift TAMs toward M1 polarization, inhibit local neovascularization signals, and the growth and metastasis of Lewis lung cancer (131). In addition, GBM promotes macrophage infiltration by up-regulating LOX, which secretes SPP1 to stimulate angiogenesis in the PTEN-knockout GBM xenograft mouse model to sustain the growth of glioma cells. However, inhibition of LOX reduces macrophage infiltration and tumor growth (25). Finally, it is found that tumor stromal infiltrating inflammatory cells, TAMs, and (MCs) mast cells synergistic promote tumor angiogenesis and promote the occurrence, invasion, and metastasis of NSCLC (132).

Infiltration of tumor tissue TAMs in patients with lung adenocarcinoma is related to tumor lymphangiogenesis, which in turn leads to a lower survival rate in patients with lung adenocarcinoma (133). Furthermore, CCL2 is produced in all types of cancers and plays a particularly important role in cancer metastasis. Lymph node metastasis-associated transcript 1 (LNMAT1), a long-chain non-coding RNA, is significantly up-regulated in bladder cancer cells to enhance the transcription of CCL2, thereby recruiting TAMs to produce VEGF-C which promotes bladder cancer-associated lymphatic metastasis of tumors. Moreover, LNMAT1 is significantly overexpressed in various types of human cancer, such as bladder, prostate, kidney, colon, lung, and liver cancer (19), which also means that LNMAT1 may be a potential therapeutic target for clinical intervention in metastatic cancer.

## The Relationship Between Tumor Cells and TAMs and Targeted Therapy

Tumor cells recruit TAMs through a variety of ways, which not only inhibit their phagocytosis, but also utilize them to maintain their growth. How to prevent tumors from manipulating macrophages is also the key to current tumor immunotherapy. Nowadays, the research of tumor macrophage targeted therapy is primarily carried out from two aspects. One is to reduce the number of TAMs in tumor tissues, and the other is to reprogram TAMs into anti-tumor macrophages. Current studies have found that targeting multiple targets in the TME can reverse the M2 phenotype of macrophages (**Table 1**).

**Table 1:** Tumor treatment by targeting TAMs.

Tumor type/Cell line	Treatment of TAMs or tumor-bearing mice	Effect	References
NSCLC	Hydroxychloroquine (HCQ)	CD8+ T cell infiltration↑ Anti-tumor CD8+ T cell↑ The ratio of M1/M2 (TAMs) macrophage ↑	(134)
NSCLC cell	Ginsenoside Rh2	The expression levels of VEGF MMP2 and MMP9 ↓ The ratio of M1/M2 (TAMs) macrophage ↑	(135)
A549	Ginsenoside Rh2	Phosphorylation of α−catenin at S641↑Wnt and hedgehog signaling↓ E - cadherin↑ vimentin↓ Cell proliferation ↓	(136)
NSCLC	miR-125b	The ratio of M1/M2 (TAMs) macrophage ↑	(137)
Lewis lung cancer cells	β-Elemene	Proliferation migration and invasion↓ EMT↓ The ratio of M1/M2 (TAMs) macrophage ↑	(138)
Cisplatin-resistant A549 and H460	dasatinib	Src CD155, and MIF↓ The ratio of M1/M2 (TAMs) macrophage ↑	(139)
A549	Water extract of ginseng	Proliferation and migration↓	(140)
Lewis lung cancer cells	HangAmDan-B	Growth of LLCs↓ The ratio of M1/M2 (TAMs) macrophage ↑	(141)
Human and mouse lung tumors	ß-catenin knockdown in TAMs	Tumor size↓ The ratio of M1/M2 (TAMs) macrophage ↑	(142)
Lewis lung cancer	Fuzheng Sanjie recipe	IL-4, IL-13, TGF-α in serum ↓ IFN-γ in serum↑	(143)
Malignant pediatric brain tumors	Humanized anti-CD47 antibody	TAMs phagocytosis↑ Tumor growth ↓	(77)
PDAC	CpG	Tumor size↓ TAMs phagocytosis↑ TAMs numbers↑ The ratio of M1/M2 (TAMs) macrophage ↑	(2)
Ovarian cancer and breast cancer	Blockade of the CD24–Siglec-10 interaction	Macrophages phagocytosis↑	(78)
MHCI^hi^ Tumor cells	Disruption of either MHC class I or LILRB1	Macrophages phagocytosis↑	(79)
An *ex vivo* tumor system	PSGL-1 antibodies	Pro-inflammatory cytokine↑ The ratio of M1/M2 (TAMs) macrophage ↑	(144)
MC38-tumor-bearing mice	TLR7/8-agonist-loaded nanoparticles	Tumor size↓ The ratio of M1/M2 (TAMs) macrophage ↑	(4)
Mice bearing TSA, MCA38, 4T1 tumors	CCL16, CpG plus anti-IL-10 receptor antibody	Tumor size↓ Metastases↓ CTL activity↑ TNF IL-12 and NO in macrophage↑ The ratio of M1/M2 (TAMs) macrophage↑	(145)
LLCs MOPC315	IFN-γ + TLR agonists	TNF-α, IL-12p40, IL-12p70 and NO in macrophage↑ IL-10 in macrophage↓ Macrophage tumoricidal activity↑	(146)
Early mammary cancers	Ferumoxytol	Tumor growth↓ caspase-3 of cancer cells↑ Liver and lung metastases↓ The ratio of M1/M2 (TAMs) macrophage↑	(147)
B16F10 tumor-bearing mice	Ferumoxytol (FMT)+TLR3 agonist poly (I:C),	Tumor proliferation↓ TNF-α and iNOS in macrophage↑ Lung metastasis↓ The ratio of M1/M2 (TAMs) macrophage↑	(148)
Mouse fibrosarcoma tumor model	TLR7 ligation/TGF-β receptor I inhibition	iNOS, CD80 and MHC class II↑ TGF-β↓VEGF↓ CD4+ CD8+, CD19+ cells as well as neutrophils infiltrating the tumor↑ The ratio of M1/M2 (TAMs) macrophage↑	(149)
K1735-M2 tumor-bearing mice	Knockout TLR4 in TAMs	TNF-α↓、VEGF↓ NF-kB activity in tumor cells↓ Tumor growth↓	(90)
MC-38 tumor-bearing mice	CB2 antagonists	Tumor progression↓	(150)
C6 and GL261 glioma intracranial and subcutaneous model	Flavonoid CH625 (CYP4X1 inhibitors)	Survival↑ Tumor burden↓ Microvessel density↓ 14 15-EET-EA, VEGF and TGF-β in the TAMs↓ The ratio of M1/M2 (TAMs) macrophage↑	(151)
NSCLC	Blockade of CCL2	Growth of primary tumors↓ Spontaneous lung metastases↓ Activated intratumoral CD8+T Cells↑ The ratio of M1/M2 (TAMs) macrophage↑	(152)
Lewis lung cancer cells	Luteolin	Phosphorylation of STAT6↓ Secretion of CCL2↓ Recruitment of monocytes↓ Migration of LLCs↓	(153)
4T1	PTEN and NHERF-1	Secretion of CCL2↓VEGF-A↓ Migration of 4T1↓ The ratio of M1/M2 (TAMs) macrophage↑	(154)
Human breast cancer cells	Zoledronic acid	Tumor growth↓ Secretion of CCL2 in MSC↓ Recruitment of macrophages↓	(155)
LLC and B16F10 tumors model	CSF1R inhibitors+ CXCR2 antagonist	MDSC recruitment↓ Tumor growth↓	(156)
Breast cancer	Class IIa HDAC inhibitor (TMP95)	Tumor burden↓ pulmonary metastasis↓ Activated intratumoral CD8+T Cells↑ Vascular density↓ The ratio of M1/M2 (TAMs) macrophage↑	(157)
Colon cancer	Antibody blockade of GM-CSF and IL-6	Metastasis↓ Tumorigenesis↓ The ratio of M1/M2 (TAMs) macrophage↑	(67)
NSCLC	Bu Fei Decoction	Tumor growth↓ IL-10 and CD206 in TAMs↓ PD-L1 in NSCLC cells↓	(128)
NSCLC	IL-33 blockade	Tumor growth↓ Tregs in TME↓ The ratio of M1/M2 (TAMs) macrophage↑	(158)
Glioma	Stat3 inhibition	Tumor growth↓ The ratio of M1/M2 (TAMs) macrophage↑	(159)
	Corosolic acid (CA)-containing liposomes	IL-10↓ TNFα↑ The activation of STAT3↓ The ratio of M1/M2 (TAMs) macrophage↑	(160)
4434 melanoma LL/2 carcinoma	Knockout ERK5 in TAMs	Tumor growth↓ The activation of STAT3↓ The ratio of M1/M2 (TAMs) macrophage↑	(161)
U373 human glioblastoma	Oleanolic acid	IL-10↓ The activation of STAT3↓ The ratio of M1/M2 (TAMs) macrophage↑	(162)
Lewis lung cancer	Resveratrol	Tumor growth↓ The activation of STAT3↓ The ratio of M1/M2 (TAMs) macrophage↑	(163)
B16F10	SOCS3 Knockout	Survival time↑ The activation of STAT3↑ TNFα↓ IL-6↓ CCL8↑ Anti-tumor metastatic effect↑	(164)
Lewis lung cancer	Gefitinib	The activation of STAT6↓ Invasion and migration of LLC cells↓ The ratio of M1/M2 (TAMs) macrophage↑	(165)
4T1	Knockdown STAT6	Tumor growth and metastasis↓ The ratio of M1/M2 (TAMs) macrophage↑	(166)
Lewis lung cancer	Imatinib	The activation of STAT6↓ The migration of LLCs↓ Pulmonary metastasis↓ The ratio of M1/M2 (TAMs) macrophage↑	(167)
Colon cancer	MK2 Knockout	Tumor growth↓ Tumor Angiogenesis↓ The ratio of M1/M2 (TAMs) macrophage↑	(168)
Ovarian cancer	Deletion of ABC transporters	Tumor progression↓ Cholesterol efflux↓	(56)
NSCLC	AXL targeting in tumor cells	ICAM1 and ULBP1 in tumor cells↑ NK and CTL-mediated killing↑ Patient survival↑	(169)
Breast cancer, colon carcinoma, Melanoma,	Antibody targeting of MARCO	Tumor growth and metastasis↓ Immune checkpoint therapy↑ The ratio of M1/M2 (TAMs) macrophage↑	(170)
PC14PE6 A549	Overexpression of surfactant protein A in tumor cells	NK cells activity↑ The ratio of M1/M2 (TAMs) macrophage↑ Lung cancer progression↓	(171)
Colorectal cancer	MFHAS1 knockdown	Tumor progression↓ Migration↓ E-cadherin↑ F-Cyclin D1 and N-cadherin↓ The activation of STAT6↓ The ratio of M1/M2 (TAMs) macrophage↑	(58)
Lewis lung cancer	Metformin	The AMPKα1 activation↑ Metastases↓ Tumor angiogenesis↓ The ratio of M1/M2 (TAMs) macrophage↑	(172)
Lewis lung cancer	Astragaloside IV	Tumor growth and metastases↓ Survival time↑ The AMPKα activation↓ The ratio of M1/M2 (TAMs) macrophage↑	(173)
Lewis lung cancer	Endostatin	Tumor growth and angiogenesis↓ VEGF↓ IL-6↓ IL-17 ↓ IFN-γ↑ HIF-1α↑ Infiltration of CD8+T cells↑ The ratio of M1/M2 (TAMs) macrophage↑	(174)
Lewis lung cancer	Qing-Re-Huo-Xue (QRHX) formulae	Tumor growth↓ Infiltration of TAMs↓ IL-6↓TNF-α↓Arg-1↓iNOS↑ VEGF↓CD31↓ CXCL12/CXCR4↓ JAK2/STAT3 phosphorylation↓	(175)
Lewis lung cancer	IFNγ and/or celecoxib	Tumor growth↓ MMP-2↓MMP-9↓VEGF↓ The density of microvessels↓ The ratio of M1/M2 (TAMs) macrophage↑	(176)
Lewis lung cancer CMT167	Knockout cPLA2	Infiltration of TAMs↓ Tumor growth and metastases↓ Survival time↑ IL-6↓	(177)
Lewis lung cancer	Melittin	Tumor growth↓ VEGF↓ The ratio of M1/M2 (TAMs) macrophage↑	(178)
Lewis lung cancer	MEL-dKLA (hybrid peptide)	Tumor growth and angiogenesis↓ The ratio of M1/M2 (TAMs) macrophage↑	(179)
CT-26 B16F10	RP-182 (synthetic peptide)	Tumor growth↓ Survival time↑ Antitumor activity of CD8+T cells↑ Conversion of from M2 (TAMs) macrophage into M1	(180)
B16F10	LTF-IC	Tumor growth↓ Survival time↑ Granzyme B↑ VEGF↓ MDSC↓ Treg↓ The ratio of M1/M2 (TAMs) macrophage↑	(181)
B16F10 4T1	High-salt diets	Tumor growth↓ IL-12p40↑ ICAM-1↑ IFN-γ↑ TNF-α↑ IL-6↓ IL-10↓ CSF-2↓ T-cell-mediated antitumor responses↑ M-MDSCs differentiate to M1 macrophage	(182)
Malignant phyllodes tumor	Maraviroc (a FDA-proved CCR5 inhibitor)	Tumor growth↓ Recruitment of monocytes↓	(21)
Lewis lung cancer	glycocalyx-mimicking nanoparticles (GNPs)	IL-12↑IL-10↓Arginase 1↓ CCL22↓ The ratio of M1/M2 (TAMs) macrophage↑ Tumor growth↓	(183)
Metastatic castration-resistant prostate cancer	Carlumab (CNTO 888)	Tumor growth=	(184)
Advanced pancreatic cancer	CCR2 inhibitor PF-04136309 +FOLFIRINOX chemotherapy	Infiltration of TAMs↓ IL-4↓IL-10↓ IL-13↓ TGF-β↓ CD4+T cell infiltration↑ CD8+T cell infiltration↑ Treg infiltration↓ Tumor growth↓	(185)
PDAC	CCR2 inhibitor, CXCR2 inhibitor +FOLFIRINOX chemotherapy	CD4+ T cell infiltration↑ CD8+ T cell infiltration↑ Tumor-infiltrating myeloid cells↓ Tumor growth↓	(186)
Colorectal cancer	CCR5 inhibitor	Anti-tumor repolarization of macrophages↑ IL-8↓VEGF↓ Tumor growth↓	(187)

### Reducing the Accumulation of TAMs in Tumor Tissues

The utilization of liposome-encapsulated clodronate treatment effectively depletes the infiltrating macrophages in the tumor thereby achieving significant inhibition of tumor growth (188). The CCL2/CCR2 axis plays a vital role in the recruitment of TAMs to tumor tissues. After knocking out CCR2 in mice, the macrophages accumulation in tumors is decreased, slowing down the tumor growth (30). In addition, IL-4 activates STAT6 to polarize macrophages to M2 and enhance their CCL2 secretion. However, luteolin inhibits the phosphorylation of STAT6, the main downstream of IL-4, reduces the expression of M2 related genes in macrophages and the secretion of CCL2, and affects the tumor tissue infiltration of M2 macrophages, thereby reducing CCL2-dependent metastasis of Lewis lung cancer cells (153). Furthermore, the expression of PTEN can reduce the secretion of CCL2 and the expression of VEGF-A in macrophages, and inhibit the migration of breast cancer cells. However, NHERF-1 can promote the expression of PTEN on the cell membrane where PTEN performs its function, and NHERF-1 and PTEN cooperate to inhibit the M2-type polarization of macrophages, in which the process is related to the reduction of macrophage CCL2 secretion, which indicates that that targeted enhancement of the expression of NHERF-1 and PTEN of TAMs may be an important strategy to reduce the secretion of CCL2. In addition, treatment with zoledronic acid (ZA) can reduce the expression of CCL2 in MSC cells, thereby reducing the recruitment of TAMs to the tumor site, thereby inhibiting tumor growth (155). However, it has also been found that CCL2 blockade does not inhibit the recruitment of TAMs, but tilts the polarization of TAMs toward M1, showing a stronger anti-tumor phenotype, and activating CD8+ T cells in the tumor to destroy the tumor cell (152). The differences in these studies may be related to the types of different tumors. In addition, these studies also verify the effectiveness of blockade CCL2 in animal models. However, carlumab (CNTO 888) is well-tolerated in phase 2 trial (NCT00992186) but does not block the CCL2/CCR2 axis or show anti-tumor activity as a single agent in metastatic castration-resistant prostate cancer (CRPC) (184). The combination with CCR2 inhibitor PF-04136309 and FOLFIRINOX chemotherapy to treat tumors in a phase Ib trial (NCT01413022) to achieve an objective tumor response, with local tumor control achieved in most patients (185), which suggest that inhibition of CCR2 can improve the effect of tumor chemotherapy. Indeed, it was found that although targeting CCR2+ TAMs or CXCR2+ TANs alone can enhance anti-tumor immunity, unfortunately, this leads to a compensatory influx of alternative myeloid subset, which result persistent immunosuppressive TME. It is worth noting that combined treatment with CCR2 inhibitors and CXCR2 inhibitors can overcome this compensatory effect, and augment anti-tumor immunity and improve response to FOLFIRINOX chemotherapy, prolonging the survival of PDAC mice (187). Therefore, targeting CCR2 and CXCR2 combined with FOLFIRINOX chemotherapy may be a very promising treatment for PDAC. Moreover, given that TANs secrete high levels of CCL2 and CCL17 to recruit macrophages. Therefore, in mouse HCC models, intratumoral injection of antibodies targeting CCL2 and CCL17 reduces the migration activity of macrophages and Treg cells, and delays tumor growth (28). Sorafenib is a drug that inhibits tumor angiogenesis. However, although sorafenib treatment reduces the tumor volume in mice, it increases the number of TANs infiltrating the tumor. More importantly, the combination therapy of sorafenib and anti-Ly6G antibody can reduce the number of TANs and neovascularization, which can further inhibit the tumor growth (28).

CSF1 participates in the recruitment of TAMs to tumor tissues and the M2 polarization process of TAMs, and the survival, proliferation, and function of TAMs depend to a large extent on the CSF1R signaling. The signaling of CSF1R depends on two ligands, CSF-1 and IL-34. Indeed, the high expression of IL-34 and CSF-1 in lung cancer tissues is intimately related to the progression of lung cancer (189), which suggests that IL-34 and CSF-1 secreted by tumor cells may activate and maintain the pro-tumor function of TAMs by interacting with the CSF1R receptor on TAMs. Importantly, further studies have found that IL-34 is highly expressed in ovarian cancer cells and tissues, which is associated with poor progression-free survival (PFS) and overall survival, while the expression of CSF-1 has not changed. Moreover, it’s worth noting that compared with CSF-1, IL-34 has a stronger affinity for CSF1R (190). It can be seen that tumor cells may secrete high levels of IL-34 to stimulate the CSF1R signaling of TAMs to promote tumor progression. Therefore, targeting IL-34 may be more effective than CSF-1 for the treatment of cancer. However, studies have shown that the utilization of CSF1R inhibitors to interfere the communication between TAM and tumor cells has not had any delay effect on tumor growth. Surprisingly, it is found that a large number of PMN-MDSC are recruited in tumor tissues after the treatment of CSF1R inhibitors with mice. Mechanistically, CSF1 secreted by tumor cells binds to the CSF1R receptor on CAFs and inhibits the secretion of CXCL1 and other chemokines by CAFs to recruit PMN-MDSCs. CXCR2 is the primary receptor for CXCL1 to function. It is worth noting that CSF1R inhibitor combined with CXCR2 antagonist intervention can block PMN-MDSC infiltration of tumor tissues, and has a strong tumor treatment effect (156). This study provides a novel strategy for tumor immunotherapy. In addition, class IIa histone deacetylase (Class IIa HDAC) inhibitor TMP95 can also induce macrophages to fight tumors. Additionally, ADM3100, a specific antagonist of the CXCR4/CXCL12 pathway, decreases the accumulation of TAMs and prevents B16 melanoma progression in mice (23).

### Reprogramming TAMs Into Anti-tumor Macrophages

#### Blocking Cytokine

The inflammatory TME is the primary cause of immunosuppression of TAMs. Thus, improving the inflammatory TME is an important strategy to restore phagocytic activity of TAMs. CSF-2 and IL6 antibody blockade can inhibit the occurrence and metastasis of colon cancer in vivo, increase the proportion of M1 macrophages in tumor tissues, and reduce the proportion of M2 phenotypic TAMs in tumor tissues (67). It has been observed that TMP95 can affect the response of human monocytes to CSF-1 and CSF-2 *in vitro*. Further studies have found that TMP95 can induce the M1 polarization of TAMs to phagocytose tumors, normalize tumor vasculature, and prevent tumor cell proliferation and lung metastasis (157). Besides, studies have found that MAPKAP Kinase 2 (MK2), which mediates the synthesis of proinflammatory cytokines, promotes TAMs to the M2 macrophage phenotype and promotes angiogenesis into tumor development, while chemical inhibitors of MK2 in macrophages inhibit M2 polarization and M2 macrophage-induced angiogenesis (168). In addition, in a phase I clinical trial (NCT01736813), CCR5 antagonists leads to TAMs anti-tumor repolarization and mitigates the tumor-promoting inflammation in CRC (186). These studies also show that improving the inflammatory TME is also the key to restoring the anti-tumor polarization of TAMs.

IL-10 secreted by TAMs promotes the expression of PD-L1 in NSCLC cells, while Bufei Decoction can reduce the expression of IL-10 and CD206 in TAMs and PD-L1 in NSCLC cells, delaying the progression of tumors in NSCLC (128), which also means Bufei Decoction reduces the proportion of M2 macrophages in TAMs in tumor tissues to delay tumor progression. In addition, IL-33 is an important carcinogen. It has been found that IL-33 can promote the growth and metastasis of colorectal cancer, breast cancer, gastric cancer, and ovarian cancer. The expression of IL-33 in patients with NSCLC is positively correlated with Ki-67 and the expression of M2 TAMs and Teg related genes. In fact, IL-33 blockade can reduce the polarization of M2 TAMs and the accumulation of Tregs in the tumor microenvironment, thereby inhibiting the growth of NSCLCs (158).

Sialic acid can promote the differentiation of MDSC into TAMs. Theoretically, inhibition of sialic acid can inhibit the differentiation of TAMs. However, intratumoral injection of sialidase in mice alone activates STAT3 in tumor myeloid cells and increased the infiltration of myeloid cells, accelerating the tumor growth. Intratumoral injection of STAT3 inhibitors also does not impede tumor growth, but it is surprising that the combination of sialiadase and STAT3 inhibitor JSI-124 can produce significant anti-tumor effects, which is related to the consumption of MDSCs at the tumor site (55). It suggests that sialidase can sensitize tumor myeloid cells to STAT3 inhibition, and dramatically enhance the anti-tumor effect of STAT3 targeted therapy.

#### TLR Receptors

Studies have shown that TLR7/8 agonists can be packaged in β-cyclodextrin nanoparticles to be transported to TAM, which can promote the conversion of macrophages from M2 to M1, promoting the antitumor of macrophages (4). In addition, treatment with CpG plus anti-interleukin-10 receptor antibody rapidly transfers TAMs from type M2 to type M1 (145). Moreover, studies have found that the activation of TLR3 alone or IFN-γ in macrophage cannot trigger the anti-tumor activity of macrophages, but the combined intervention of the two can enhance the M1 polarization of macrophages to activate the tumor-killing activity of macrophages (146), which also means that the combined use of IFN-γ and TLR3 agonists may be useful for macrophage-based cancers immunotherapy. Furthermore, nano-iron oxide particles promote the M1 polarization of macrophages (147). Meanwhile, nano-iron oxide particles combined with TLR3 agonists can also induce M1-type polarization of macrophages against tumors (148). The combination with activating TLR7 and inhibiting TGF-β signaling can reprogram TAMs to the M1 phenotype, which can enhance the tumoricidal activity of TAMs and reduce tumor progression (149). Altogether, these results indicate that the activation of TLR7, TLR8, and TLR9 of TAMs can change macrophages from a tumor-promoting effect to an anti-tumor effect.

The TLR4 signaling in TAMs can promote tumor growth. Indeed, blockade of TLR4 in TAMs with antibody reduces the production of cytokines and weakens their pro-tumor activity (90). However, it has been found that the expression of macrophage monoacylglycerol lipase (MGLL) in tumor tissues is reduced, and the lack of monoacylglycerol lipase (MGLL) in TAM can lead to triglyceride lipid overload. Mechanistically, the cannabinoid receptor CB2 can interact with TLR4 and inhibit the pro-inflammatory signaling of TLR4, while lack of MGLL promotes CB2/TLR4-dependent the M2 phenotype in TAMs, thereby inhibiting the function of tumor-related CD8+ T cells and promoting tumor progression. MGLL can inhibit CB2 cannabinoid receptor-dependent tumor progression, and CB2 antagonist treatment can delay tumor progression in mice (150), which indicates that TLR4 activation can also inhibit tumor growth. The specific mechanism still remains to be explored. In addition, MGLL is the switch of CB2/TLR4 dependent macrophage activation. Restoring the expression of MGLL in tumor tissues and targeting CB2 are new strategies for tumor immunotherapy. However, the flavonoid CH625 inhibits CYP4X1 through the CB2 and EGFR-STAT3 axis to reprogram TAMs away from M2 type, and normalizes the blood vessels of gliomas (151). This also indicates that CB2 can inhibit tumor progression, which may be different from tumor types and the specific mechanism deserves further exploration.

#### STAT3 and STAT6

Tumor cells induce and maintain the M2 phenotype of TAMs in many ways. Nowadays, there are also studies to reprogram TAMs by targeting to block the internal signal molecules of TAMs and restore their anti-tumor activity (**Table 1**). It is found that inhibiting the expression of STAT3 reprograms TAMs into M1 macrophages to inhibit tumor growth (159). Indeed, liposomes are used to carry corosolic acid (CA), a natural compound that inhibits stat3, to target CD163+ macrophages, thereby reprogramming TAMs to the M1 phenotype and promoting the expression of pro-inflammatory factors (160). The activation of the STAT3 signal is related to ERK5. ERK5 belongs to the family of mitogen-activated protein kinases (MAPKs) and plays an important role in the process of extracellular transduction. It has been found that high expression of ERK5 in human TAMs promotes tumor growth. Mechanistically, ERK5 phosphorylates Tyr705 of STAT3, thereby promoting the M2 phenotype of TAMs. However, knockout ERK5 in TAMs reduces the phosphorylation of STAT3 and inhibits its M2 phenotype, which promotes the anti-tumor activity of TAMs, thereby inhibiting the melanoma growth (161), which suggests that targeting ERK5 is an attractive cancer treatment strategy. It is worth noting that the activation of STAT3 is essential for the M2 differentiation of macrophages. Studies have found that oleanolic acid, a triterpene compound, can inhibit the activation of STAT3 in macrophages and glioblastoma cells to suppress the M2 phenotype differentiation of macrophages and the proliferation of glioblastoma cells (162). In addition, resveratrol inhibits the growth of lung cancer by inhibiting the M2-like polarization of TAMs and inhibiting the activation of STAT3 in tumor cells (163). Indeed, STAT3 is also frequently activated in a variety of human cancer cells, STAT3 signaling transduction promotes the growth and survival of tumor cells. However, studies have found that mice whose macrophages specifically knock out the SOCS3 that has been shown to be a negative regulator of STAT3 survive longer than wild-type mice. Mechanistically, tumor lysates stimulated SOCS3 knockout macrophages in vitro, their STAT3 phosphorylation is enhanced and TNF-α and IL-6 are reduced, and higher levels of MCP2/CCL8 via STAT3 are produced to combat tumor metastasis in contrast to normal macrophages. Therefore, inhibition of SOCS3 activity in macrophages may have a therapeutic effect on inhibiting tumor metastasis (164). These results also indicate that whether the activation of STAT3 in macrophages promotes tumor development remains to be clarified.

STAT6 phosphorylation is important signaling for M2-like polarization of macrophages. It has been found that gefitinib can inhibit IL-13-induced phosphorylation of STAT6 in macrophages to inhibit M2-like polarization in the Lewis lung cancer model (165). Indeed, targeting the STAT6 in TAMs can reduce the growth and metastasis of breast cancer tumors (166). Furthermore, imatinib inhibits STAT6 phosphorylation and nuclear translocation, leading to the arrest of M2-like polarization of macrophages and inhibiting the metastasis of Lewis lung cancer (167). Furthermore, glycocalyx-mimicking nanoparticles (GNPs) can be specifically internalized by TAMs via lectin receptors, resulting in to up-regulation IL-12 and down-regulation of IL-10, arginase 1, and CCL22 expression, reversing TAMs to an anti-tumor phenotype, in which the process is associated with suppressing STAT6 and activating NF-κB phosphorylation. Importantly, the combined treatment of GNPs and PD-L1 antibody can significantly improve the immunosuppressive TME and the efficacy of anti-tumor therapy (183). These studies have shown that targeting STAT6 can suppress the M2 polarization of TAMs and delay tumor growth.

#### Polypeptide

Melittin, a major polypeptide of bee venom, has been extensively studied due to its cytotoxicity to malignant cells. The melittin selectively reduces the number of CD206+ M2-like TAMs in the lewis lung cancer mouse model, which is manifested in the decreased expression of VEGF and CD206 in M2-like TAMs (178). However, the effect of melittin in the treatment of lung cancer is limited. Therefore, studies have utilized melittin’s affinity for M2 type macrophages and mixed melittin with pro-apoptotic peptide d (KLAKLAK)2 (dKLA) to target TAMs in the lung cancer tissue. Indeed, MEL-dKLA is more able to inhibit tumor growth rate, tumor weight, and angiogenesis compared with melittin (179). In addition, RP-182, the host defense peptide, can also reprogram M2-like TAMs to an antitumor M1-like phenotype, and selectively induce a conformational switch of CD206 on M2-like TAMs, thereby increasing the cancer cell phagocytosis by TAMs (180). Furthermore, previous studies have found that the immune complex of lactoferrin has a very strong pro-inflammatory effect on human monocytes and macrophages (191). Indeed, the immune complex of lactoferrin drives the transformation of human macrophages from M2- to M1-like phenotype (192), and also mediates anti-tumor effects by resetting TAMs to the M1-like phenotype (181). In addition, iron-loaded tumor-associated macrophages (iTAMs) exhibit a pro-inflammatory phenotype, which has anti-tumor activity and the ability to reduce tumor growth in patients with lung adenocarcinoma, and prolong the survival time of patients. However, the iron in TME does not affect the survival of the patients in patients with lung squamous cell carcinoma, indicating that iron has a unique effect on different histological subtypes of NSCLC (193). The specific mechanism needs to be explored.

#### High Salt Diets

High-salt diets can also encourage macrophages to swallow tumors to slow the growth of tumors. Recent studies have found that high-salt diets inhibit tumor growth in mice by regulating the differentiation of bone marrow-derived cells. The local sodium chloride concentration in tumor tissues results in high osmolality, which reduces the production of cytokines required for the expansion of MDSCs and the accumulation of MDSCs in the blood, spleen, and tumors. Therefore, two types of MDSCs change their phenotypes: monocytes-MDSCs differentiate into anti-tumor macrophages, and granulocytes-MDSCs adopt pro-inflammatory functions, thereby reactivating the anti-tumor actions of T cells (182). Although high-salt diets have pro-inflammatory and anti-tumor effects, it has been found that high-salt diets can induce increased production of pro-inflammatory cytokines, and activate p38 MAPK and STAT1 signaling pathways to promote immune activation of DC cells and accelerate the progression of SLE in mice (194). These results also suggest that a high-salt diet is also a double-edged sword. It can cause excessive inflammation while treating tumors, which may induce autoimmune diseases.

#### Target Other Signaling Molecules

Tumor cell-derived HA drives cholesterol efflux from macrophages to induce their TAMs phenotype, so knocking out the ABC transporter which mediates cholesterol efflux can reverse the tumor-promoting function of TAMs and reduce tumor progression (56). Scavenger receptor MARCO is expressed in tissue-resident macrophages in the lung, lymph nodes, spleen, and peritoneum. In addition, it has been found that targeting MARCO expression in M2 TAMs in mouse breast cancer, melanoma, and colon cancer can increase the infiltration of M1 type macrophages in tumor tissues, reduce M2 type macrophages, and block tumor growth and metastasis (170). Moreover, metformin also inhibits the M2-like polarization of TAMs, which is associated with the enhanced level of AMPKα1 phosphorylation in macrophages. Similarly, another AMPK activator, AIRCA, can prevent IL-13-stimulated M2-like polarization. However, knocking out AMPKα1 has no effect. In addition, metformin can inhibit the pro-angiogenesis of M2-like macrophages to prevent tumor metastasis (172). However, astragaloside IV blocks the M2 polarization of macrophages by inhibiting the AMPK signaling pathway of macrophages and reduces the growth, invasion, migration, and angiogenesis of lung cancer cells. Astragaloside IV inhibits AMPKα activation in M2 macrophages, but silencing AMPKα partially abolishes the inhibitory effect of AS-IV (173). Therefore, the effect of AMPK activation on macrophage polarization remains unclear.

Malignant fibrous histiocytoma amplified sequence 1 (MFHAS1) is a predicted oncogenic protein that shows tumorigenic activity *in vivo*. It is worth noting that CRC cells can induce the expression of MFFAS1 in mouse macrophages. However, knocking down the expression of MFHAS1 in macrophages reduces the number of M2 TAMs and delays the progression of CRC (58), which also means that MFFAS1 in macrophages is also one of the most potential targets for tumor immunotherapy. The expression of TREM-1 on TAMs decreases significantly with tumor growth, which is a feature of lung cancer TAMs. In addition, TREM-1 activation can promote TAM to secrete IL-1β in the intervention of LPS (195). This also indicates that the low expression level of TREM-1 in TAMs may affect the pro-inflammatory effect of TAMs, which is beneficial to tumor growth. Complement component 9 (C9) plays a critical role in killing tumors *in vitro*. C9 is specifically expressed in most alveolar macrophages (AMs) in adjacent lung tissues, but only a few tumor TAMs in NSCLC tissues. More importantly, the percentage of C9 positive cells in AMs or TAMs is related to the increase in tumoricidal activity. In fact, the hypoxic tumor microenvironment can shift the phenotype of macrophages from M1 to M2 and down-regulate the expression of C9 in TAMs (196), which means that improving the hypoxic environment of tumors is an important strategy for tumor treatment. Surfactant protein A (SP-A) is a large multimeric protein that exists in the airways and alveoli of the lung. In addition to performing immunomodulatory functions in infectious respiratory diseases, SP-A can also be used as a marker for lung adenocarcinoma. The deletion of SFTPA1 (alias, SPA) gene in NSCLCs is associated with tumor progression. However, overexpression of SP-A in lung adenocarcinoma cells can inhibit the progression of lung cancer by promoting the M1 polarization of TAMs to recruit and activate NK cells (171). These studies also provide new potential targets for tumor immunotherapy.

#### Target Interaction Receptors on TAMs and Tumor Cells

After intervention therapy with anti-CD47 antibody, macrophages can be induced to phagocytose tumor cells (77). However, CD47-blocking as a monotherapy shows limited anti-tumor effects in the pancreatic ductal adenocarcinoma (PDAC) model, which may be related to the limited phagocytosis of macrophages after CD47 blockade. Fortunately, it has been found that CpG oligodeoxynucleotide, a toll-like receptor agonist, can directly activate TLR9 to trigger an innate immune response. In addition, its stimulation can cause changes in the central carbon metabolism of macrophages to enhance the anti-tumor activity of macrophages (2). It is worth noting that mitochondrial DNA (mtDNA) contains a large number of unmethylated CpG sequences (197). It is speculated that treatment with mtDNA to target macrophages in tumors may enhance their anti-tumor activity. In addition, therapeutic blockade of the CD24 and SIGLEC10 interaction and the MHC class I molecules and LILRB1 interaction accompanied with enhanced TAM phagocytosis towards tumor cells (78, 79). The expression of PD-1 in TAMs is negatively correlated with the phagocytic ability against tumor cells. Blockade of PD-1/PD-L1 axis *in vivo* can increase macrophage phagocytosis, reduce tumor growth, and prolong the survival time of mice tumor model (80). These results mean that interfering with the interaction of these targets between tumor cells and macrophages can provide new therapeutic strategies for tumor immunotherapy. Furthermore, the latest research found that PSGL-1 is a new macrophage checkpoint. Importantly, targeting PSGL-1 with an antagonist antibody can repolarize M2 macrophages to M1 type (144).

### Inhibition of Tumor Angiogenesis

Endostatin has been proven to effectively inhibit the angiogenesis and growth of endothelial cells. Endostatin can reduce the number of M2 macrophages and increase the number of M1 macrophages in tumor tissues, and promote the infiltration of CD8+ T cells into tumors. In addition, endostatin can reduce the expression levels of VEGF, TGF-β, IL-6, and IL-17 in tumor tissues, increase the expression levels of IFN-γ and HIF-1α, suppressing growth and angiogenesis of tumors (174). Besides, QRHX intervention can reduce the infiltration of TAMs in tumor tissues of lung cancer mice, reduce the expression of IL-6, TNF-α, ARG-1, CD31, and VEGF, and increase the expression of inducible NO synthase (iNOS). It is worth noting that CXCL12/CXCR4 and JAK2/STAT3 are important molecules involved in M2 polarization. QRHX also reduces the expression of CXCL12/CXCR4 and the phosphorylation of JAK2/STAT3 in tumor tissues (175). These results also indicate that QRHX may induce the conversion of M2 type macrophages to M1 type macrophages, reducing tumor tissue angiogenesis. Besides, IFNγ and/or celecoxib, the cyclooxygenase-2 inhibitor, treatment constantly suppress tumor growth in mouse lung cancer models. Mechanistically, IFNγ or celecoxib increases the percentage of M1 macrophages and decreases the percentage of M2 macrophages in tumors. In addition, they also reduce the density of MMP-2, MMP-9, VEGF, and microvessels in tumors (176). Furthermore, the IL-6 secreted by tumor cells and stromal cells contributes to tumor progression and angiogenesis, but the expression of cPLA2 in macrophages is critical to the release of IL-6 from tumor cells. The loss of cytoplasmic phospholipase A2 (cPLA2) in macrophages can reduce the secretion of IL-6 by tumor cells and prevent the progression and metastasis of lung cancer (177), which indicates that cPLA2 plays a key role in tumor progression, and targeting the expression of cPLA2 in TAMs can provide new therapeutic strategies for the clinical treatment of tumors.

## Summary

There is a complex crosstalk between tumor cells and TAMs in tumors. Macrophages have the function of engulfing and killing tumor cells. However, a large number of TAMs infiltrated in the tumor microenvironment not only kill tumor cells, but also support tumor growth and metastasis. Specifically, first, tumor cells secrete a large number of chemokines to recruit macrophages into tumor tissues, such as CCL2, CCL3, CCL5, and so on. Then, after the macrophages recruited to the tumor tissue, the tumor cells secrete a variety of cytokines to regulate the signaling pathway of the macrophages, and then promote their differentiation into M2 macrophages. Finally, TAMs differentiated into M2 type maintain tumor growth by promoting tumor cell growth, invasion, and metastasis, inhibiting the killing of tumor cells by immune cells, and angiogenesis in tumor tissues. Importantly, these processes are intimately related to the inflammatory TME. How to regulate the inflammatory TME is the key to improving the immunosuppressive TME. Moreover, the large number of bacteria present in tumor tissues may be an important cause of the inflammatory TME. Therefore, targeting microbes in tumors may be the key to restore tumor phagocytosis of TAMs. There are also numerous corresponding strategies to limit the supporting effect of TAMs on tumor growth. The main strategy is to limit the recruitment of monocytes, which reduces the accumulation of TAMs in tumor tissues, and target TAMs receptors or block key cytokines secreted by tumor cells to reprogram TAMs into M1 macrophages with anti-tumor activity. Because a single antibody targeting TAMs therapy may cause other immune cells to compensately infiltrate the tumor tissue, and then continue to maintain the inflammatory TME, there are certain limitations. In the future, multiple methods should be considered to target TAMs. For example, multi-site targeted combined drug therapy or a suitable high-salt diet may have a better therapeutic effect. In addition, the current clinical trials on TAMs-targeted treatments in animals is still in a lagging stage, and further improvement is urgently needed. It is worth noting that recent studies have found that RXRβ is a new marker on the surface of TAMs. Targeting RXRβ can distinguish TAMs from macrophages in other tissues (116). Similarly, it is found that a new type of bivalent glycosylated targeting ligand packaging nanoparticles can utilize CD206 to selectively target anti-inflammatory M2 macrophages (198). These studies provide new drug carrier tools and targeted markers for targeting M2 macrophages. Nowadays, the drug resistance and heterogeneity of cancer stem cells are the fundamental reasons for the failure of radiotherapy, chemotherapy, and immunotherapy. How to eliminate cancer stem cells in tumor tissues is of great importance. In addition, recent studies have concluded that the growth of cancer stem cells is extremely dependent on methionine (199). These research results provide crucial treatment ideas and strategies for cancer treatment.

## Author Contributions

ZG is responsible for the retrieval of relevant documents and the writing of article content, and SD is responsible for the design of the overall framework of the article. All authors contributed to the article and approved the submitted version.

## Funding and Acknowledgments

This work was supported by grants from the National Natural Science Foundation of China (No. 31671241).

## Conflict of Interest

The authors declare that the research was conducted in the absence of any commercial or financial relationships that could be construed as a potential conflict of interest.
